# MRL proteins cooperate with activated Ras in glia to drive distinct oncogenic outcomes

**DOI:** 10.1038/onc.2017.68

**Published:** 2017-03-27

**Authors:** E Taylor, N Alqadri, L Dodgson, D Mason, E Lyulcheva, G Messina, D Bennett

**Affiliations:** 1 Department of Biochemistry, Institute of Integrative Biology, University of Liverpool, Liverpool, UK; 2 Centre for Cell Imaging, Biosciences Building, University of Liverpool, Liverpool, UK

## Abstract

The Mig10/RIAM/Lpd (MRL) adapter protein Lpd regulates actin dynamics through interactions with Scar/WAVE and Ena/VASP proteins to promote the formation of cellular protrusions and to stimulate invasive migration. However, the ability of MRL proteins to interact with multiple actin regulators and to promote serum response factor (SRF) signalling has raised the question of whether MRL proteins employ alternative downstream mechanisms to drive oncogenic processes in a context-dependent manner. Here, using a *Drosophila* model, we show that overexpression of either human Lpd or its *Drosophila* orthologue Pico can promote growth and invasion of *Ras*^*V12*^-induced cell tumours in the brain. Notably, effects were restricted to two populations of Repo-positive glial cells: an invasive population, characterized by JNK-dependent elevation of Mmp1 expression, and a hyperproliferative population lacking elevated JNK signalling. JNK activation was not triggered by reactive immune cell signalling, implicating the involvement of an intrinsic stress response. The ability to promote dissemination of *Ras*^*V12*^-induced tumours was shared by a subset of actin regulators, including, most prominently, Chicadee/Profilin, which directly interacts with Pico, and, Mal, a cofactor for serum response factor that responds to changes in G:F actin dynamics. Suppression of Mal activity partially abrogated the ability of *pico* to promote invasion of *Ras*^*V12*^ tumours. Furthermore, we found that larval glia are enriched for serum response factor expression, explaining the apparent sensitivity of glial cells to *Pico/Ras*^*V12*^ overexpression. Taken together, our findings indicate that MRL proteins cooperate with oncogenic Ras to promote formation of glial tumours, and that, in this context, Mal/serum response factor activation is rate-limiting for tumour dissemination.

## Introduction

Regulation of actin-based structures is critical for normal cell adhesion, morphology and motility.^1^ Correspondingly, aberrant cytoskeletal dynamics are implicated in the motility and dissemination of cancer cells.^2, 3^ In addition to the direct effects of actin reorganization, for example on lamellipodia-like structures at the leading edge of invasive cells,^4^ regulators of cytoplasmic actin also control the localization and activity of myocardin-related transcription factors (MRTF/Mal), which are transcriptional coactivators of serum response factor (SRF), by regulating the availability of monomeric (G-)actin.^5^ Depletion of nuclear and cytoplasmic G-actin in response to increased actin polymerization increases the rate of MRTF/Mal translocation to the nucleus, reduces the rate of nuclear export of MRTF/Mal and derepresses the expression of genes that require MRTF/Mal for transcription, leading to SRF-dependent transcription.^6, 7, 8^

The Mig-10/RIAM/Lamellipodin (MRL) family of adapter proteins transduce signals derived from growth factor receptors, via interactions with Ras-like GTPases and/or phospholipids, to changes in the actin cytoskeleton, increased lamellipodia protrusion, cell motility and altered cell adhesion.^9, 10^ Effects on the actin cytoskeleton are mediated by direct interactions with various actin regulatory proteins, including Ena/VASP, Scar/WAVE and Profilin.^9, 10, 11^ MRL proteins are also capable of activating SRF signalling by altering the ratio of G:F actin.^12^ MRL proteins are therefore good candidates for genes that drive tumour cell invasion and metastasis. Indeed, in breast cancer, Lpd is upregulated in tumours with lymph node metastases compared to lymph node-negative tumours^13^ and also in highly invasive MDA-MB231 breast cancer cells compared to non-invasive MCF7 breast cancer cells or normal breast tissue.^14^ Furthermore, increased expression and membrane localization correlate with reduced metastasis-free survival and poor prognosis in breast cancer patients.^15^ Mechanistically, MRL proteins promote invasive 3D breast cancer cell migration via interactions with the actin regulators Scar/Wave and Ena/VASP.^15^ Lpd is also part of the ‘Ras cancer signature’ as it is upregulated in human breast epithelial cells transformed with oncogenic Ras.^16^ The ‘Ras signature’ reflects the activation status of the Ras pathway and has been successfully used to identify patterns of pathway deregulation in human tumours and to identify clinically relevant associations with disease outcomes.^16^ An understanding of the functional consequences of MRL–Ras interactions in cancer development is, however, currently lacking.

*Drosophila* encodes only one MRL protein, called Pico, enabling the dissection of conserved cancer promoting effects of the MRL gene family in an animal model, with the potential to help guide studies in mammalian systems.^17^ Many biological processes related to tumorigenesis and metastasis are well conserved in flies and nearly all of the genes linked to cancer progression in humans are present in the *Drosophila* genome.^17, 18^ Here we have tested the prediction that MRL proteins might cooperate with oncogenic Ras by promoting invasiveness of Ras^V12^-induced tumours in the larval eye disc and brain. Notably, we observed tumour overgrowth and invasion, but these cooperative effects were restricted to cells expressing the pan-glial marker Repo; loss of overexpression in glia, and not in other cell types, completely suppressed oncogenic cooperation. Notably, SRF is strongly enriched in glia providing an explanation for why glia were specifically affected. Moreover, overexpression of *mal*, a cofactor for SRF, or *chickadee, Drosophila* profilin, also cooperated strongly with oncogenic Ras to drive glial invasion. Taken together, our findings provide experimental evidence for the role of MRL proteins in the hyperproliferation and transformation of glial tumours *in vivo*. Furthermore, Profilin and downstream SRF signalling predominantly drive this process rather than other MRL-interactors, Ena/VASP and Scar/WAVE, as is the case in other contexts.

## Results

### Pico cooperates with oncogenic Ras to promote tumour dissemination

**Figure 1:**
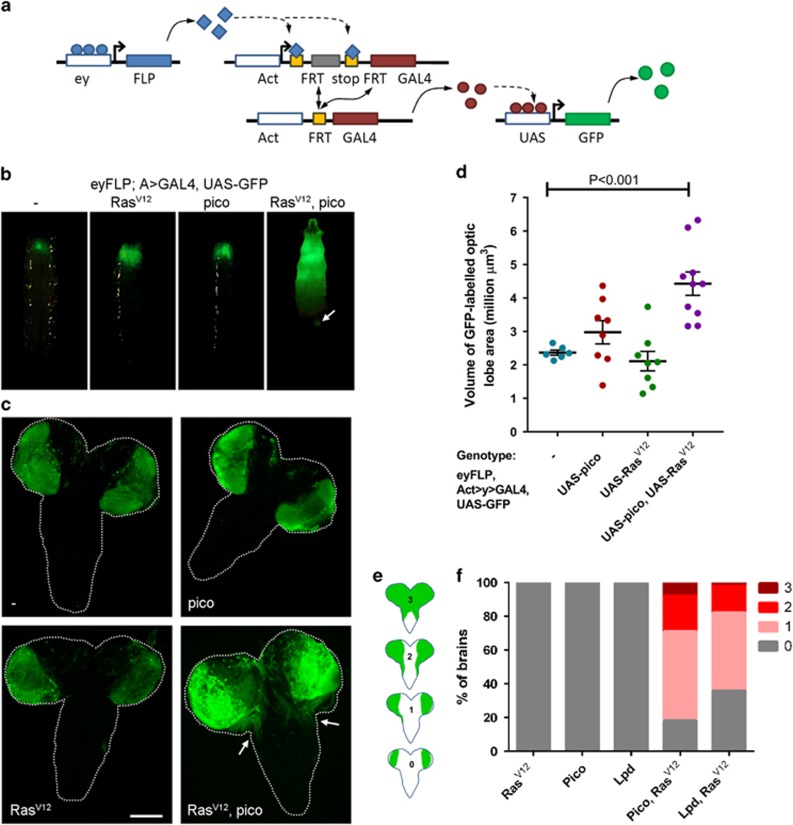
Pico promotes spreading of Ras^V12^-induced tumours. (**a**) Schematic outlining heritable overexpression of UAS transgenes following expression of *eyFLP* and removal of an *FRT*-flanked linker from *Act>GAL4* reconstituting the *Act-GAL4* driver. This driver then constitutively drives expression of *UAS-GFP* and other UAS constructs in daughter cells. (**b**) Images of whole larvae showing distribution of GFP expression induced in the eye-discs and optic lobes of larva of different genotypes, as indicated. Expression of GFP alone or together with the transgenes indicated was driven by flipping-out an FRT-flanked linker from an Act>GAL4 element using eyFLP (eyFLP, Act>GAL4). Overexpression of *Ras*^*V12*^ with *pico* resulted in a dramatic increase in GFP-marked tissue sometimes leading to the formation of GFP foci at more distant sites (arrow). (**c**) Distribution of GFP expression in dissected brains showing overgrowth of the optic lobe and invasion of GFP-labelled cells into the VNC in *Ras*^*V12*^*, pico* brains (VNC, arrows). Scale bar 100 μm. (**d**) Quantification of the volume of GFP-labelled cells in the optic lobes of the indicated genotypes, based on optical sections taken throughout the entire brain. Mean value of individual data points±s.e., is indicated. (**e**, **f**) Quantification of the invasion phenotype. (**e**) Individual cephalic complexes were assigned to one of four categories, depicted, based on the degree of VNC invasion observed: *Type 0,* no invasion of the VNC, *Type I*, tumour cell invasion occurring down one side of the VNC, *Type II,* tumour cells invading both sides of the VNC; and, *Type III,* significant tumour cell invasion of the VNC combined with overgrowth/fusion of the optic lobes. (**f**) Stacked bar chart showing the percentage of brains expressing either *Ras*^*V12*^, *pico, hLpd, pico/Ras*^*V12*^ or *hLpd/Ras*^*V12*^, classified into each of the four categories (*n*=100 brains/genotype).

Oncogenic mutations in Ras are frequent events in early stages of cancer development, driving proliferative overgrowth and contributing to tumour formation. The Ras pathway also modulates cytoskeleton organization, cell motility and expression of metastasis signature genes,^19^ but cooperation between oncogenic Ras and its downstream targets are poorly understood. To test the interaction between Pico and Ras, we used a cancer model in *Drosophila* in which genetically defined tumours can be induced in the developing eye disc and brain.^20, 21^ In this model (Figure 1a), expression of the *Flp* gene, under the control of the *eyeless* promoter (*eyFLP*), is used to irreversibly switch on constitutive, GAL4-mediated expression of upstream activator element (*UAS*) target genes in the developing eye. This is achieved by FLP-mediated recombination between two Flp recombination target (*FRT*) sites flanking a linker cassette that otherwise silences the *Actin-GAL4* (*ActGAL4*) driver. Once induced, GAL4 binds to and drives the expression of *UAS*-containing transgenes.

Using this approach, we examined the effect of overexpressing *pico* or *Ras*^*V12*^ alone or together in GFP-labelled cells in the eye imaginal discs and optic lobes of wandering third instar larvae. Notably, coexpression of *pico* and *Ras*^*V12*^ led to an accumulation of GFP-labelled cells and redistribution to more distant sites. This effect was not observed when either gene was overexpressed in isolation (Figure 1b). To quantify the effects on tissue overgrowth we captured images of optical sections through brains dissected from the different genotypes and measured the volume occupied by GFP-labelled cells. There was no significant difference in volume of GFP-labelled cells expressing *pico* or *Ras*^*V12*^ alone compared to controls (Figures 1c and d). In contrast, *pico* and *Ras*^*V12*^ co-overexpression resulted in a 1.9-fold increase in volume of GFP-labelled cells in the optic lobes compared to GFP alone controls, *P*<0.001 (Figure 1d).

Inspection of the distribution of GFP-labelled cells in the brain revealed that GFP-labelled *pico*/*Ras*^*V12*^ tumour cells had invaded into the ventral nerve cord (VNC) in the majority (82/100) of cases, whereas cells expressing *pico* or *Ras*^*V12*^ alone never extended beyond the optic lobe (Figure 1c). To quantitate the tumour cell invasion phenotypes produced for each of the genotypes, brains were assigned to one of four categories based on the degree of VNC invasion observed: Type 0, no invasion of the VNC; Type I, tumour cell invasion occurring down one side of the VNC only; Type II, tumour cells invading both sides of the VNC; Type III, significant tumour cell invasion of the VNC combined with fusion of the optic lobes (Figure 1e). Cephalic complexes dissected from animals expressing *pico* and *Ras*^*V12*^ were entirely composed of Type 0 brains, whereas only 18% of *Ras*^*V12*^*/pico* brains were found to exhibit no VNC invasion. Fifty-three percent of *Ras*^*V12*^*/pico* brains were found to have mild Type I invasion, and 21 and 8% of brains were assigned to Type II and Type III categories, respectively (Figure 1f). To test functional conservation, we examined the effect of ectopic overexpression of human Lpd (hLpd) in this system. Brains expressing hLpd showed no evidence of invasion, but, like *pico*, hLpd was able to drive invasion of Ras^V12^-induced tumours, which occurred in 64/100 of cases (Figure 1f).

We previously showed that *pico* promotes coordinated growth and proliferation in the wing imaginal discs^12^ prompting us to wonder whether other promoters of tissue growth could also drive the dissemination of otherwise benign Ras^V12^ tumour cells into neighbouring tissues. To address this, we tested the effects of co-overexpressing *Drosophila cyclin-D* (*cycD*) and *cyclin-dependent kinase-4* (*cdk4*) in our assay. There was no significant difference in volume of GFP-labelled cells in optic lobes expressing *Ras*^*V12*^ with or without overexpressed *cycD* and *cdk4*, and GFP-labelled cells were never located outside of the eye-antennal discs/optic lobe region (Supplementary Figure S1). This is in agreement with previous reports that proliferative cues such as *cycD* and *cdk4* do not account for presence of tumour cells in the VNC.^21^

### Invasive Pico/Ras^V12^ tumours are characterized by elevation of Mmp1 and extracellular matrix remodelling

**Figure 2:**
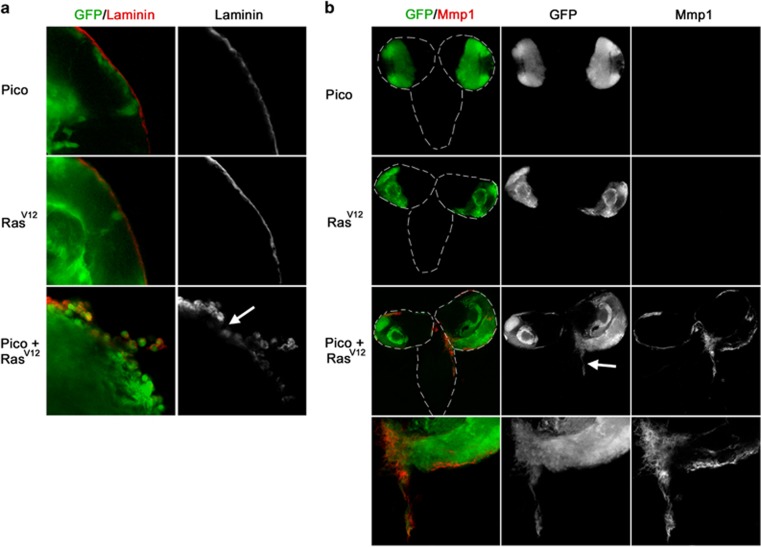
Brains coexpressing *Ras*^*V12*^ and *pico* display extracellular matrix degradation and ectopic expression of Mmp1. (**a**) Optic lobes from larvae overexpressing *pico*, *Ras*^*V12*^ or *Ras*^*V12*^*, pico* under the control of eyFLP, Act>GAL4, stained with anti-Laminin antibody, which labels the surface of the optic lobes. Laminin staining was found to be severely interrupted in brains coexpressing *Ras*^*V12*^ and *pico* but not from brains expressing *pico* or Ras^V12^ alone. (**b**) Distribution of the metalloproteinase Mmp1. Little or no Mmp1 staining was observed in animals expressing *Ras*^*V12*^ or *pico* alone. In contrast, animals co-overexpressing *Ras*^*V12*^ and *pico* had elevated Mmp1 around the edges of the optic lobes and at sites of invasion into the VNC (arrow).

Degradation of the extracellular matrix by matrix metalloproteases (MMPs) is required during tissue remodelling and during the progression of many types of cancer.^22, 23^ To investigate integrity of the extracellular matrix, we examined the distribution of Laminin, which is a major component both of the basement membrane underpinning the basal side of epithelial cells and of the gliovascular basal lamina of the blood brain barrier. In brains ectopically expressing either *pico* or *Ras*^*V12*^, Laminin staining of the optic lobes was found to be smooth and uninterrupted. In contrast, discontinuous Laminin staining was observed around the optic lobes of *Ras*^*V12*^/*pico* brains, consistent with degradation of the extracellular matrix (Figure 2a). When we examined MMP expression we found that Mmp1 was found to be largely absent in brains overexpressing either *pico* or *Ras*^*V12*^. In contrast, a marked increase in Mmp1 levels was observed in cephalic complexes expressing both *pico* and *Ras*^*V12*^(Figure 2b). Interestingly, Mmp1 expression was not detected in all *Ras*^*V12*^*, pico* cells; Mmp1 staining was mainly observed in the marginal regions of the optic lobes and in the tumour cells that had invaded the VNC (Figure 2b).

### JNK activation is required for Pico/Ras^V12^-mediated MMP expression and tumour cell spreading

**Figure 3:**
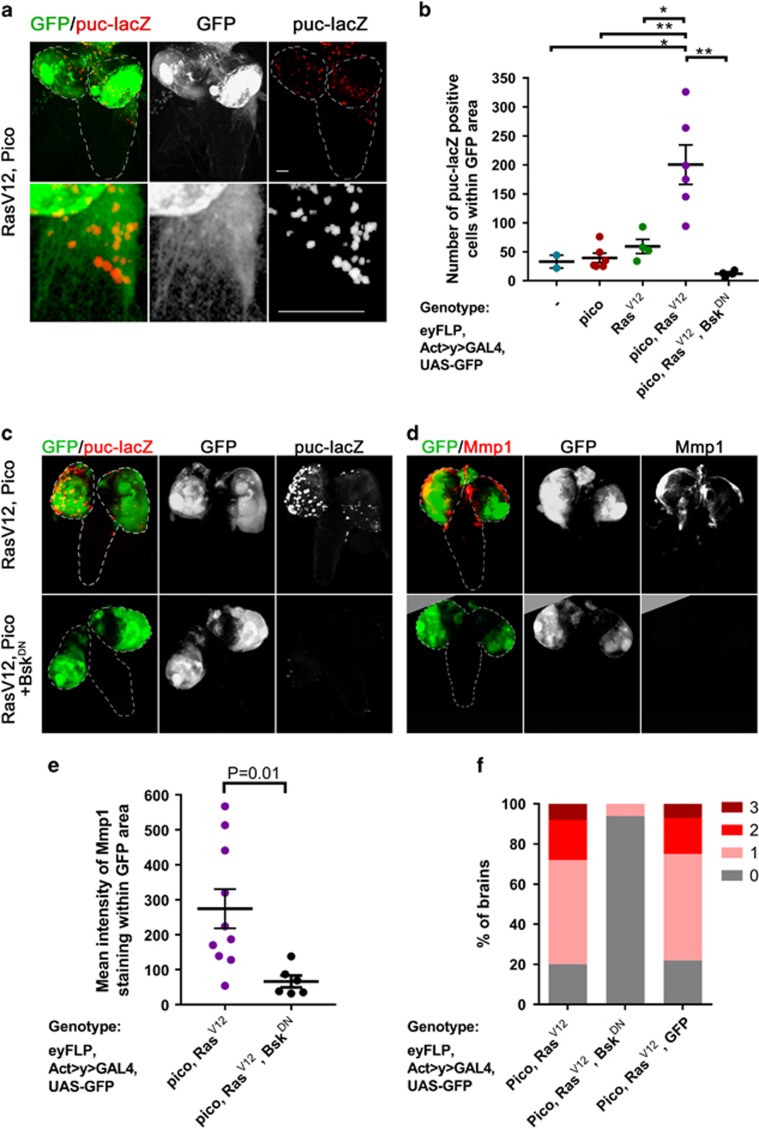
Mmp1 accumulation and invasion into VNC is dependent on JNK activation. (**a**) Co-overexpression of *Ras*^*V12*^ and *pico* in GFP-labelled tumours (green) leads to JNK activation in some tumour cells, based on β-galactosidase staining to detect *puc-lacZ* (red). Top panels show low magnification images of brains, lower panels show magnified images of tumours invading into VNC, which are enriched in puc-lacZ staining. Scale bars, 100 μm. (**b**) Quantitation of number of puc-lacZ positive foci in GFP-labelled areas of the optic lobes from the indicated genotypes. Mean value of individual data points±s.e., is indicated. (**b**, **c**) Blockade of JNK activation with dominant-negative Bsk/JNK (*Bsk*^*DN*^) suppresses activation of the JNK pathway. (**c**) Representative images showing puc-lacZ induction in *Ras*^*V12*^ *pico* tumours and suppression of this effect by *Bsk*^*DN*^: the top row of images were taken from representative *Ras*^*V12*^ *pico* larval brains; the bottom row were taken from siblings coexpressing *Bsk*^*DN*^. (**d**, **e**) Blockade of JNK activation with dominant-negative Bsk/JNK (*Bsk*^*DN*^) suppresses the induction of Mmp1. (**d**) Representative images of *Ras*^*V12*^ *pico* tumours with or without *Bsk*^*DN*^. (**e**) Quantitation of mean intensity of Mmp1 within GFP-labelled *Ras*^*V12*^ *pico* tumours in the presence or absence of *Bsk*^*DN*^. Mean value of individual data points±s.e., is indicated. (**f**) *Bsk*^*DN*^ suppresses *Ras*^*V12*^*/pico*-mediated invasion into the VNC, whereas an additional ‘inert’ UAS element (UAS-GFP) does not. Graph summarizing extent of invasion in the different genotypes (*n*=100 brains of each type) according to the scale introduced in Figure 1, with 3 being the most severe and 0 corresponding to no invasion.

Studies of *Ras*^*V12*^ tumours with impaired cell polarity (for example due to mutations in the tumour suppressor gene *scrib*) have revealed that JNK activation is critical for Mmp1 upregulation and tumour cell invasion of the VNC.^24^ To assess the state of JNK signalling in *Ras*^*V12*^*/pico brains*, we monitored the levels of *puckered*, a downstream target of JNK (Martín-Blanco *et al.*^25^) using a *lacZ* enhancer trap (*puc-lacZ*). We observed limited *puc-lacZ* staining in brains expressing *Ras*^*V12*^ or *pico* alone, but in *Ras*^*V12*^*/pico* brains we observed a significant increase in the number of *puc-lacZ*-positive nuclei (*P*<0.01) indicative of elevated JNK activation in these cells (Figures 3a and b). Not every cell showed *puc-lacZ*-positive nuclei indicating that JNK activation was not a necessary outcome of *Ras*^*V12*^*/pico* overexpression (Figure 3a).

To determine the requirement for JNK signalling in *Ras*^*V12*^*/pico*-mediated metastasis we tested the effect of coexpressing a dominant-negative form of the *Drosophila* JNK, encoded by *basket* (*bsk*^*DN*^). Ectopic overexpression of *bsk*^*DN*^ strongly suppressed JNK activation as monitored with *puc-lacZ* (Figures 3b and c). Strikingly, overexpression of *bsk*^*DN*^ also reduced Mmp1 levels 4.1-fold (*P*<0.01) in GFP-labelled tumour cells (Figures 3d and e) and also almost completely blocked *Ras*^*V12*^*/pico-*mediated tumour cell invasion of the VNC (Figure 3f). In the absence of *bsk*^*DN*^, evidence of spreading was observed in 80/100 cases of *Ras*^*V12*^*/pico* tumours, whereas in siblings coexpressing *bsk*^*DN*^, invasion was only evident in 6/100 cases (Fisher’s exact test, *P*<0.0001). Using our scale of the extent of invasion (Figure 1d) the average stage score of invasion in *Ras*^*V12*^*/pico* larvae was 1.16±0.08 (mean ±s.e.m.), but this was significantly reduced by coexpression of *bsk*^*DN*^ to 0.06 ±0.02, (Student’s *t*-test, *P*<0.001). The effect of *bsk*^*DN*^ was not due to titration of GAL4 in these experiments because substitution of the *UAS-bsk*^*DN*^ element with *UAS-GFP* restored the invasive capability of *Ras*^*V12*^*/pico* (Figure 3f). Taken together, these data indicate that *pico* cooperates with oncogenic Ras to promote JNK activation, and that JNK activation is essential for invasion of *Ras*^*V12*^*/pico* tumours.

### TNF-mediated immune response is not required for pico/Ras^V12^-mediated invasion

**Figure 4:**
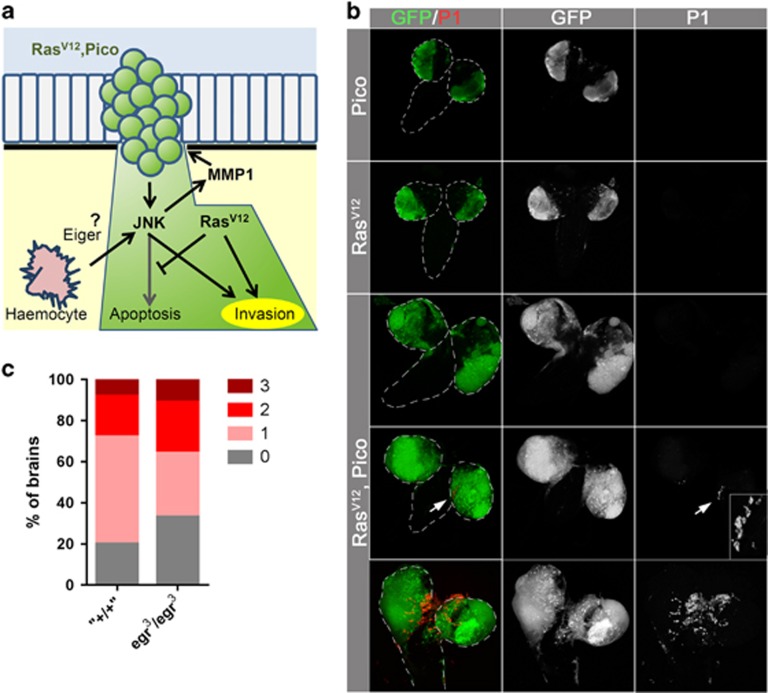
Invasion is not driven by haemocyte recruitment and TNF/Eiger-dependent signalling. (**a**) Possible model of extrinsic signalling from haemocytes to JNK activation in tumours. In the absence of *Ras*^*V12*^, activated JNK leads to cell death, whereas in its presence, cell death is suppressed and JNK promotes Mmp1 expression. Consequently, JNK and *Ras*^*V12*^ cooperate to drive tumour cell invasion. TNF/Eiger-secreting haemocytes that are recruited to sites of certain primary tumours, for example *scrib*^*−/−*^ *Ras*^*V12*^, have been reported to be capable of providing extrinsic cues that trigger JNK activation, raising the possibility this is also the case for *pico Ras*^*V12*^ tumours. (**b**) Images of dissected brains showing distribution of haemocytes, as detected with an anti-NimC1 P1 antibody. Haemocytes were not detected in brains expressing *pico* or *Ras*^*V12*^ alone. In *pico* or *Ras*^*V12*^ brains, haemocytes were sometimes observed at sites of invasion, but this was not a necessary outcome. (**c**) Graph summarizing extent of invasion in the different genotypes (*n*=100 brains of each type).

Accumulating evidence suggests that diversion of host immunity can contribute to the acquisition of invasive behaviour. In *Drosophila*, inflammatory responses, mediated by Eiger/TNF-producing haemocytes, trigger JNK activation leading to invasive behaviour of Ras^*V12*^-induced tumours^26^ (Figure 4a). The mechanism of haemocyte recruitment to tumours is not well understood. To test the involvement of the immune response in *Ras*^*V12*^*/pico* brains, we examined whether there was accumulation of haemocytes at sites of tumour invasion. Although we observed haemocyte recruitment to a proportion of *Ras*^*V12*^*/pico* brains, haemocyte number was not correlated with presence or severity of cellular invasion; some invasive tumours lacked associated haemocytes (Figure 4b). To test whether *pico*-mediated metastasis is driven by diversion of the host immune response mediated by TNF/eiger, we tested the effect of *Ras*^*V12*^/*pico* overexpression in an *eiger* null genetic background. Loss of *eiger* modestly suppressed invasion in *Ras*^*V12*^*/pico* brains (Figure 4c); whereas 80/100 *Ras*^*V12*^*/pico* animals showed GFP-labelled cells in the VNC, invasion was observed in 67/100 *Ras*^*V12*^*/pico* animals lacking *eiger* (*eiger*^*3*^*/eiger*^*3*^), which was at the borderline of significance (Fisher’s exact test, *P*=0.054). However, there was little effect on the average stage of invasion, which reduced from 1.16±0.08 to 1.14±0.10 (Student’s *t*-test, *P*=0.88) when *eiger* was absent. Taken together this indicates that *pico* does not primarily promote invasive behaviour through diversion of a TNF-mediated immune response.

### Pico cooperates with activated Ras to drive distinct oncogenic outcomes in glia

**Figure 5:**
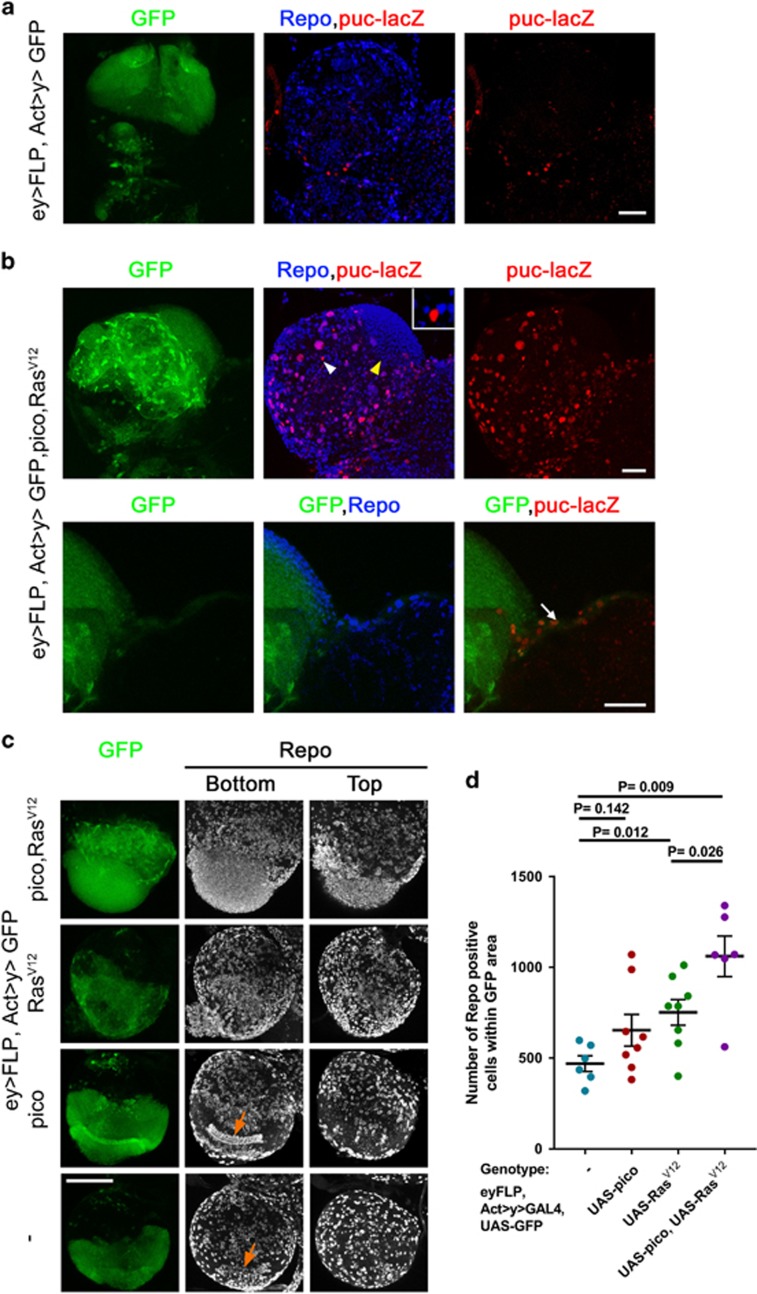
Distinct oncogenic effects in glial populations. (**a**, **b**) Optic lobes from third instar larvae, orientated with the VNC to the right of each image. GFP-expressing regions are in green, glia are marked with Repo in blue and JNK activation is marked with *puc-lacZ* in red. (**a**) GFP-only expressing control showing that reconstitution of Actin-GAL4 after flipping out an FRT-flanked linker with eyFLP drives GFP expression heritably in glial lineages marked with Repo, as well as other lineages. Some cells are also labelled with puc-lacZ, although the majority of these lie outside the GFP-labelled area. (**b**) Coexpression of *Ras*^*V12*^ and *pico* results in two distinct effects observable in Repo +ve glia (top panels): activation of JNK, marked with *puc-lacZ* expression, and accumulation of glial cells in a region proximal to the VNC (yellow arrowhead), largely lacking *puc-lacZ* expression. Inset is a magnified image of a Repo –ve cell staining positive for *puc-lacZ* (white arrowhead). Magnified images (bottom) show a GFP and *puc-lacZ* labelled population that has invaded into the VNC (arrow). Scale bars, 50 μm. (**c**) Anti-Repo staining showing the effect of *Ras*^*V12*^ and *pico* co-overexpression on glial distribution and number in optic lobes. Repo-stained images are 2D projections of confocal z-stacks from the bottom and top of the same optic lobe. Bottom sections reveal stereotypical arrangement of glia (arrows) in control optic lobes and those overexpressing *pico*, which is lost upon expression of *Ras*^*V12*^. Scale bar, 100 μm. (**d**) Graph showing quantification of number of Repo-positive glia in GFP-labelled areas of the optic lobes from the indicated genotypes. Mean value of individual data points±SE is indicated.

When we looked at distribution of JNK activation more closely in *Ras*^*V12*^ *pico* brains it was apparent that many puc-lacZ positive cells decorated the surface of the optic lobes. This non-random distribution made us wonder whether JNK activation was restricted to specific cell types. Indeed, we found the combination of *eyFLP* with *AyGAL4* was capable of driving expression in a range of cells in the larval optic lobes including glia (Figure 5a), consistent with previous reports showing expression in neuroblasts, lamina and medulla neurons, neurophils and medulla cortex glia.^27^ When we looked at the distribution of neuronal and glial markers in *Ras*^*V12*^ *pico* tumours we found that 96±3% of puc-lacZ positive cells in GFP-labelled tumours also stained for the pan-glial marker Repo (*n*=4). GFP-labelled cells invading into the VNC were of this type (Figure 5b, arrow); puc-lacZ staining in these cells is consistent with our genetic data indicating a requirement for JNK to mediate Mmp1 expression and extracellular matrix breakdown (see Figure 3). Although rare, we did observe a few Repo-negative GFP-labelled cells with puc-lacZ staining, although interestingly these were typically juxtaposed directly next to Repo-positive glial cells (Figure 5b, white arrowhead and inset, see Discussion). We also observed a distinct Repo-positive population consisting of many small cells that were puc-lacZ negative, located in a region of the optic lobe that had appeared to have overproliferated (Figure 5b, yellow arrowhead; Figure 5c). When we counted the number of Repo-positive cells in GFP-labelled tumours within the optic lobes (Figures 5c and d), we found that *Ras*^*V12*^ overexpression led to a 1.6-fold increase in glial number in GFP-labelled regions compared to GFP only controls (*P*<0.01). The increase in glial number was not matched by a significant increase in GFP-labelled tumour volume (Figure 1), most likely because many *Ras*^*V12*^-overexpressing cells were small,^28^ suggesting *Ras*^*V12*^-expressing glia move more quickly through the cell cycle without an accompanying increase in mass. Co-overexpression of *pico* significantly enhanced the effect of *Ras*^*V12*^ (Figure 5d), leading to a 2.3-fold increase in glial number compared to GFP alone controls (*P*<0.05). Taken together, the data above indicate that ectopic *Ras*^*V12*^ and *pico* cooperate to promote overproliferation of one glial cell population in the developing optic lobe without the activation of JNK, while promoting JNK activation and cell invasion in another glial population.

### Overexpression of Ras^V12^ Pico in glia is necessary for an increase in tumour volume and cell invasion

**Figure 6:**
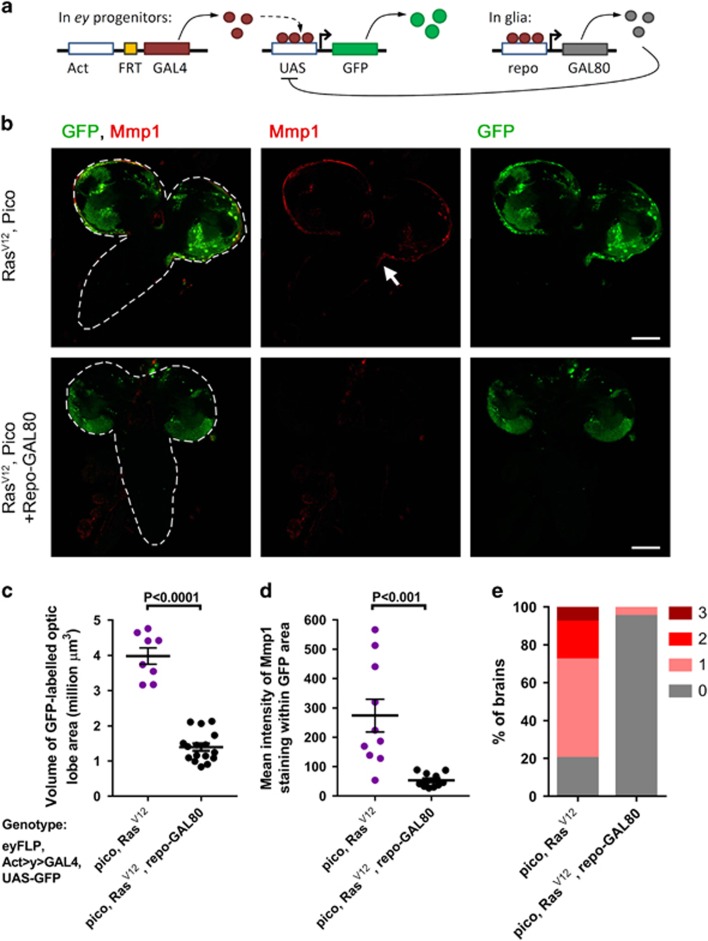
Transcriptional blockade in glia blocks cooperation between *Ras*^*V12*^ and *pico*. (**a**) Schematic showing suppression of GAL4-mediated overexpression in glia using *repo-GAL80*. (**b**) Brains (outlined with dashed line in first set of panels) overexpressing *Ras*^*V12*^, *pico* with or without *repo-GAL80*, showing distribution of GFP-labelled cells (green) and Mmp1 (red). Animals co-overexpressing *Ras*^*V12*^, *pico* displayed invasion of Mmp1-expressing cells into the VNC (arrow). Mmp1 staining and invasion were suppressed in siblings containing *repo-GAL80*; optic lobes of these animals were also reduced in size. Scale bars, 100 μm. (**c**) Measurements of the volume of GFP-labelled *Ras*^*V12*^, *pico* optic lobe tumours with or without *repo-GAL80*. (**d**) Measurements of mean intensity of Mmp1 staining in *Ras*^*V12*^, *pico* tumours with or without *repo-GAL80*. (**c**, **d**) Mean value of individual data points±SE is indicated. (**e**) Stacked bar chart summarizing extent of invasion in the different genotypes (*n*=100 brains of each type).

To test if the tumour overgrowth and invasion phenotypes we had observed in the optic lobe were due to ectopic expression of *Ras*^*V12*^ and *pico* in *eyFLP*-expressing Repo^+^ glia, we repeated our experiments in a *repo-GAL80* background to block GAL4-mediated expression specifically in *repo*-positive glia but not in other cell types (Figure 6a). When we measured the volume of GFP-labelled tumours in *Ras*^*V12*^*/pico* optic lobes from animals with (*n*=8) or without *repo-GAL80* (*n*=16), we found that *repo-GAL80* reduced the mean tumour volume 2.9-fold (*P*<0.001; Figure 6c). The mean intensity of Mmp1 staining in GFP-labelled tumours was also reduced 5.1-fold (*P*<0.001; Figure 6d). Correspondingly, there was a significant reduction in the instances of invasion into the VNC to 5/100 of cases (Fisher’s exact test, *P*<0.0001) and a corresponding reduction in the average stage score of invasion to 0.05±0.02 (*P*<0.0001; Figure 6e). As an additional test, we further validated these findings by using a more restricted *eyeless*-driven FLPase, *ey(3.5)FLP*, which does not drive substantive expression in the optic lobes of the brain,^29^ (Supplementary Figure S2). Overexpression of *Ras*^*V12*^/*pico* with *ey(3.5)FLP* did not replicate the growth and invasion phenotypes observed with *eyFLP*, consistent with our observations that overexpression in glia was required (Supplementary Figure S2). Expression of *Ras*^*V12*^ specifically in GFP-labelled glia with *repo-GAL4* was pupal lethal but led to overgrowth and extension of the larval VNC (mean VNC length 132% of control, Student’s *t*-test *P*<0.05, *n*=10). Coexpression of *Ras*^*V12*^ with *pico* led to lethality at the wandering larval stages and extension of the VNC was significantly enhanced (to 187% of control, *P*<0.01, *n*=9), again consistent with a cooperative interaction in glia (Supplementary Figure S3). As part of the ‘Ras signature’, Lpd is implicated to act downstream of oncogenic Ras in human breast epithelial cells prompting us to test this possibility in our system. *pico* knockdown by RNAi did not significantly modify the extension of larval VNC exhibited in brains overexpressing *Ras*^*V12*^ (*P*>0.05, Supplementary Figure S3), so we conclude that *pico* is not limiting for *Ras*^*V12*^ in this context.

### SRF is enriched in larval glia in the CNS

**Figure 7:**
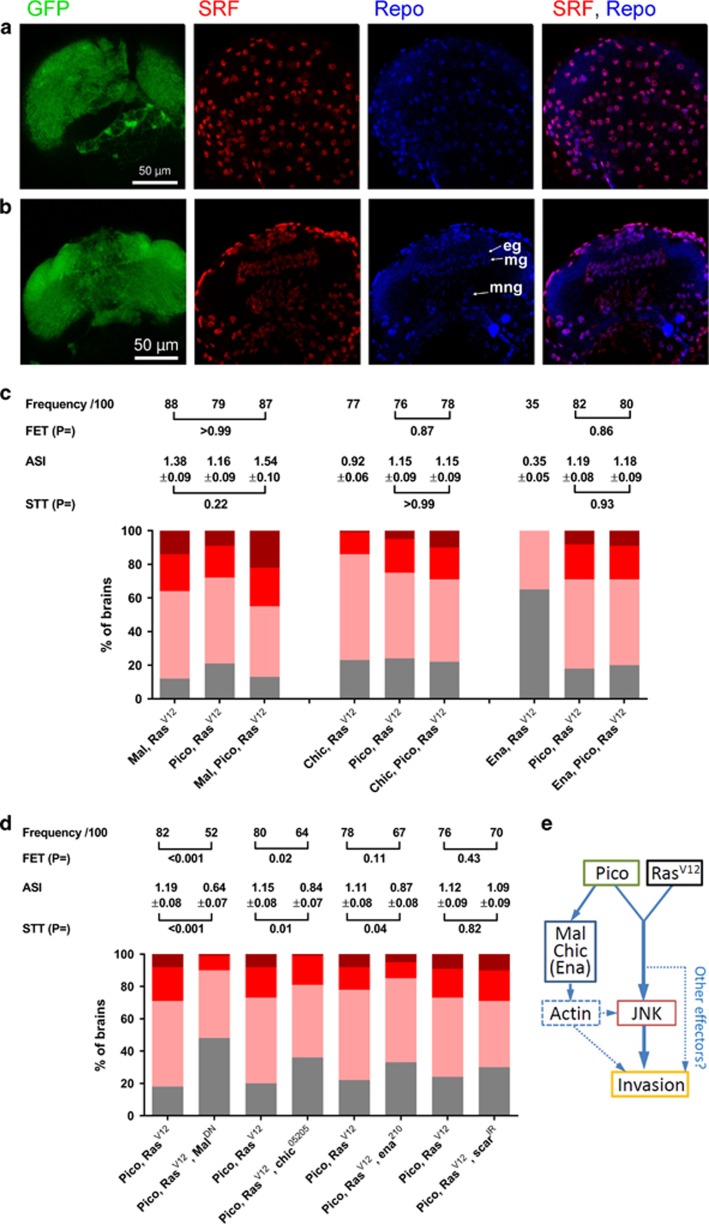
Mal and Chic are rate limiting for tumour dissemination. (**a**, **b**) Distribution of SRF in the third instar larval optic lobes. SRF antibody staining (red) overlaps that of Repo (blue) both at the surface (**a**) of the optic lobes and in cross-section (**b**). In cross-section, staining in epithelial (eg), marginal (mg) and medulla neurophil (mng) glia is evident.(**c**) Cooperation between *Ras*^*V12*^ and cytoskeletal regulators results in cancer cell invasion into the VNC. Stacked bar charts summarizing extent of invasion in the different genotypes (*n*=100 brains of each type) according to the scale introduced in Figure 1, with 3 being the most severe and 0 corresponding to no invasion. Shown above each group of charts is a summary of statistical tests for key pairwise combinations, as indicated, in each experiment. Frequency represents number of brains showing invasion/total number (100 in each case); FET, Fisher’s exact test; ASI, average stage score of invasion; STT, Student’s *t*-test. (**d**) *mal*^*DN*^ and *chic* loss of function partially suppress the effect of *Ras*^*V12*^ *pico* co-overexpression. *ena* and *scar* loss of function have reduced or no effect, respectively. Results are grouped by sibling pairs (overexpressed *pico Ras*^*V12*^±genetic modifier) and are displayed as in (**c**). (**e**) Model of cooperation between Pico and *Ras*^*V12*^ co-overexpression. Pico and *Ras*^*V12*^ cooperate to activate JNK, which is necessary for invasion of glia from the optic lobes into the VNC. Mal, Chic, and to a lesser extent Ena, are rate limiting for invasion. These regulators may contribute directly or indirectly to invasion via changes in the actin cytoskeleton (see Discussion).

Why should glia be particularly sensitive to coexpression of ectopic *pico* and *Ras*^*V12*^? We recently demonstrated that overexpression of *pico* reduces the ratio of G:F-actin and is capable of inducing activation of SRF signalling *in vitro*.^12^ This prompted us to question whether the cooperation between *pico* and *Ras*^*V12*^ was mediated by SRF signalling. Although SRF is expressed throughout the adult brain,^30^ where it plays roles in sleep and visual memory,^30, 31^ we wondered whether SRF expression is spatially regulated in the CNS earlier in development, as it is in other tissues such as the wing imaginal disc (data not shown). Notably, we detected strong anti-SRF antibody staining in glia from third instar optic lobes (Figures 7a and b). SRF staining was evident both in Repo positive surface glia (Figure 7a) and in other glial types (Figure 7b).

### The ability of Pico to promote tumour invasion is shared by selected actin regulatory genes

To further examine the contribution of actin dynamics and SRF to the development of invasive cell behaviours, we tested the effect of co-overexpressing oncogenic *Ras*^*V12*^ together with Profilin/*chic*, which has multiple roles in the augmentation of F-actin dynamics, or with regulatory proteins that bind Pico and are known to control actin polymerization by affecting the number of free barbed ends: Enabled/*ena* (anti-capping factor) and SCAR (actin nucleation).^32^ We also tested the effect of ectopic *mal*, which encodes a cofactor for SRF and responds to changes in actin dynamics to induce SRF-dependent gene expression. Overexpression of any of the above factors alone in the absence of *Ras*^*V12*^ did not induce invasive behaviour as determined by the lack of GFP-positive cells in the VNC. However, overexpression of *mal*, *chic*, *ena* or *scar* was sufficient to promote the acquisition of invasive behaviour in otherwise benign *Ras*^*V12*^-expressing tumours (Figure 7c). Based on the percentage number of larvae showing GFP-labelled cells in the VNC, these overexpression constructs can be ranked according to their invasive potential in this system, as follows: *mal* (88%)> *pico* (79%)> *chic* (77%)> *ena* (35%)> *scar* (25%), where percentage of larvae with invasion into the VNC are shown in parenthesis (*n*=100 in each case). This is also in agreement with the average stage of invasion for *Ras*^*V12*^-induced tumours coexpressing these regulators: *mal* (1.38±0.09)> *pico* (1.16±0.09)> *chic* (0.92±0.06)> *ena* (0.35±0.05)> *scar* (0.25±0.04). When we tested the effect of coexpressing *Ras*^*V12*^ and *pico* together with either *mal, chic* or *ena* we found the degree of invasion observed was not significantly increased compared to the effect of pairwise combinations of these inducers or pico alone with *Ras*^*V12*^ (Figure 7c). The lack of an additive effect suggests that these proteins may act in the same pathway to induce invasion, albeit to different extents.

MRL proteins interact directly with Profilin, Ena/VASP and the Scar/Wave complex via a number of proline-rich regions present in their C-terminal regions.^9, 10, 11, 12, 33^ To test whether these regions of Pico might be necessary for promoting invasion of Ras^V12^ tumours, we expressed a truncated version of *pico* encoding only its central RA-PH domain (*pico*^*RA-PH*^). *pico*^*RA-PH*^ failed to promote cell invasion into the VNC alone or together with coexpression of Ras^V12^ (0/100 cases of invasion in each case), suggesting that physical interaction between Pico and its downstream effectors are important for cooperation with oncogenic Ras. To explore the requirement of *chic*, *ena* and *scar* for invasion driven by *Ras*^*V12*^ *pico* we combined *Ras*^*V12*^ *pico* with the following loss-of-function alleles or RNAi: *chic*^*05205*^, *ena*^*210*^ and *scar* RNAi (*scar*^*IR*^*, VDRC-21908*). Notably, *chic* dominantly suppressed the ability of *Ras*^*V12*^ *pico* to drive invasion when one copy of the gene was mutated (Figure 7d), significantly reducing both the number of cases of tumour invasion in siblings (from 80/100 to 64/100 cases; Fisher’s exact test, *P=*0.02) and the average stage of invasion from 1.15±0.08 to 0.84±0.07 (Student’s *t*-test, *P*=0.01). Loss of one copy of *ena* had a more modest effect; there was not a significant reduction in the number of cases of invasion (*P*=0.11) but the stage of invasion was significantly reduced (*P*=0.04). *scar* knockdown did not significantly suppress invasion of *Ras*^*V12*^ *pico* cells into the VNC (Figure 7d). Notably, the same line of *scar* RNAi was observed to suppress the effects of *pico* overexpression on developmentally regulated invasive border cell migration,^11^ consistent with the idea that there are context-dependent mechanisms by which MRL proteins drive invasion.

Profilin, Ena/VASP and the Scar/Wave complex affect the actin cytoskeleton directly but are also capable of promoting Mal-SRF activity via altered actin dynamics. To assess the likely contribution of direct versus indirect effects on the actin cytoskeleton, we tested the effect of a dominant-negative version of Mal (*mal*^*DN*^), which lacks its C-terminal transcription activation domain.^34^ Compared to *Ras*^*V12*^*/pico* control animals, there was a significant reduction in the number of cases of tumour invasion in siblings coexpressing *mal*^*DN*^ (82/100 to 52/100 cases, respectively; Fisher’s exact test, *P*<0.0001). There was also a significant reduction in the average stage of invasion, from 1.19±0.08 to 0.64±0.07 (Student’s *t*-test, *P*<0.0001) when *mal*^*DN*^ was present (Figure 7d). Taken together this indicates that indirect effects via Mal/SRF signalling are rate-limiting for invasion of *Ras*^*V12*^*/pico* tumours (Figure 7e).

## Discussion

Here, we find that *pico* overexpression is capable of promoting distinct oncogenic behaviours in *Ras*^*V12*^-induced tumour cells. In particular, we observed an invasive cell population showing elevation of JNK signalling, and a hyperproliferative population lacking JNK activation. These effects were restricted to glia since the affected cell populations labelled positively with the pan-glial marker, Repo, and cooperation between *Ras*^*V12*^*/pico* was lost upon transcriptional repression in glia with *repo-GAL80*. In glia, JNK is likely to act as a proapoptotic signal as it does in epithelia—indeed, subperineurial glial cells possess a cryptic JNK-dependent apoptotic programme.^35^ However, any such programme must be suppressed by survival signals from oncogenic Ras as it is in other contexts.^36^ We found that Mmp1 expression was JNK-dependent, supporting the idea that JNK activation is subverted by tumour cells to promote invasion. Haemocytes were not always observed at sites of invading *Ras*^*V12*^*/pico* tumours, although we cannot rule out that they had not been present prior to the point at which we dissected samples for analysis. Nevertheless, invasion was not significantly affected by complete loss of *eiger*/TNF, which is a key haemocyte-secreted cytokine capable of eliciting immune responses, including JNK activation, in tumour cells.^26^ One possibility is that transformed glial cells may be resistant to haemocyte attachment and/or signalling. Examination of the cell type-specific expression pattern of TNF signalling components, such as the recently identified TNF/Eiger receptor Grindelwald,^37^ may provide a mechanistic explanation for why glia respond differently from epithelial tumour cells to circulating immune cells. Alternatively, transformed glia may express inhibitory cell surface or secreted molecules making them refractory to the innate immune system, as is the case for human glioma cells.^38^

Interestingly, a small number of Repo negative cells overexpressing *Ras*^*V12*^/*pico*, adjacent to Repo positive *Ras*^*V12*^/*pico* tumour cells, also displayed elevated JNK activity. In addition to roles in CNS development and function, glia are considered to be primary immune cells of the CNS that survey the CNS for neuronal damage, modulating inflammatory responses and engulfing debris or foreign material.^39^ The JNK pathway mediates glial engulfment activity in *Drosophila*,^40, 41^ raising the intriguing possibility that *Ras*^*V12*^*/pico* stimulates glial phagocytosis of tissue damage caused by premalignant tumour cells. Diversion of the glial damage response programme by carcinoma cells has previously been reported in murine organotypic brain slice co-cultures,^42^ stimulating local invasion in tumours resistant to glial-induced apoptosis. It will therefore be interesting to examine whether this phenomenon is JNK-dependent.

Recent work has shown the actin cytoskeleton acts both upstream and downstream of JNK^43, 44, 45^ and, conceptually, changes in cell tension resulting from altered actin cytoskeleton may trigger JNK as part of a stress response. We were interested to explore whether actin regulators that associate with Pico could similarly cooperate with *Ras*^*V12*^. In breast cancer cells, the ability of Lpd to promote 3D invasion relies on its interactions with both Ena/VASP and Scar/WAVE.^15^ Although both *ena* and *scar* were capable of cooperating with *Ras*^*V12*^ in our model, their effect was modest compared to the effect of *chic* (*Drosophila* Profilin). This might be because Ena and Scar are not limiting, or it might reflect a specific requirement for Chic, which was also limiting for the effect of *pico/Ras*^*V12*^. Interestingly, in this regard, Profilin assists in coordination of actin turnover,^46, 47^ which is the driving force for membrane protrusion and spreading of some types of glia in the CNS.^48^ Recent work has also demonstrated that changes in actin dynamics driven by MRL proteins and their binding partners can activate SRF signalling.^12, 49^ Several lines of evidence suggest that Mal/SRF signalling is important for *pico*/*Ras*^*V12*^ cooperation: first, SRF expression is enriched in glia; second, the effects of overexpression of *mal* were at least as potent as those of *pico*; third, *mal*^*DN*^ suppressed the *pico*-mediated invasion of *Ras*^*V12*^-induced tumours. In mammalian cells the majority of SRF target genes encode cytoskeletal components^50^ and recent work in *Drosophila* suggest that actin itself is a key homeostatic target.^51^ Control of Mal/SRF activity therefore may provide a mechanism by which cytoskeletal gene expression is coordinated with cytoskeletal regulation.

In summary, our data indicate that overexpression of MRL proteins is capable of driving invasion and overproliferation of Ras^V12^-induced glial cell tumours in an *in vivo* experimental model. Notably, our findings, in glia, implicate *Drosophila* Profilin and SRF signalling in MRL-mediated tumour dissemination, whereas interactions between Lpd and Ena/VASP and Scar/WAVE have been reported to be critical in the invasion of breast cancer cells. This points to important differences in the mechanism of action of MRL proteins depending on the cellular context. Given that SRF is capable of promoting human glioma cell migration^52^ and Lpd overexpression has been detected in glioma samples from patients,^53^ investigation into whether Lpd or SRF levels are associated with disease progression and patient outcome is warranted.

## Materials and methods

### Fly husbandry and genetics

Flies were reared at 25 °C under standard conditions. All initial *Drosophila* strains have been previously described. Third instar larvae were examined 6 days after egg laying. Genotypes are provided in a supplementary file.

### Immunohistochemistry

Tissues dissected from third instar larvae were fixed and stained as Ciurciu *et al.*,^54^ with minor modifications. After fixation for 20 min in 4% (w/v) paraformaldehyde in PBS, dissected brains from third instar larvae were washed in PBS with 0.1% Triton-X (PBST), then blocked for 2 h in PBST with 5% FCS (blocking solution). Primary antibody staining was done overnight at 4°C in blocking solution, washed three times with PBST and incubated with secondary antibody in blocking solution for 2 h at room temperature. After three washes in PBST, brains were mounted in Vectasheild mounting media (Vector laboratories, Peterborough, UK). Primary antibodies were as follows: rabbit anti-Laminin (1:1000); guinea-pig anti-Repo (1:1000); rabbit anti-Repo (1:25,000); mouse anti-NimC1 P1^55^(1:30), mouse anti-Mmp1 (1:1:1 mix of 3A6B4, 3B8D12, 5H7B11 from Developmental Studies Hybridoma Bank, Iowa, USA diluted 1:10); mouse anti-β−gal (Promega, Southampton, UK, 1:100); mouse anti-SRF (Active Motif, La Hulpe, Belgium, 1:100). Secondary antibodies were conjugated to Alexa-Fluor 555 or 633 (Invitrogen, Paisley, UK, 1:500). TO-PRO-3 Iodide (Invitrogen, 1:1000) or DAPI was used to visualize DNA.

### Image acquisition and analysis

Dissected tissues were imaged on a Leica MZ10F stereomicroscope for scoring of invasion phenotypes, which was done blinded, or on Zeiss LSM710, 780 or 880 microscopes equipped with 405 nm, 488 nm, 568 nm and 633 nm lasers using either Fluor × 20 or Plan Apochromat × 40/1.3NA oil immersion objective. Confocal images were imported into OMERO^56^ and adjusted for brightness and contrast uniformly across entire fields where appropriate. Figures were constructed in Adobe Photoshop. Quantitative analysis of raw confocal data was conducted using Imaris version 8.2.0 (Oxford Instruments/Bitplane, Zurich, Switzerland). The GFP channel was segmented into 3D volumes (5 μm surface grain size) by absolute intensity using an automatically selected intensity threshold. To remove small unattached objects, only the two largest volumes were kept per experiment (corresponding in all cases to the optic lobes), and their volume measured. To count the number of Repo or puc-lacZ positive cells, the above volumes were used to mask the relevant intensity channel, which was then subject to spot segmentation using an estimated spot diameter of 5 μm and background subtraction. Spots were subjected to an automatically thresholded intensity filter. All automatic thresholding was visually inspected and adjusted if necessary. For quantitation of Mmp1 staining, stacks were projected in ‘z’ and then background subtracted in the Mmp1 channel. The GFP channel was used to segment, then the selection was measured in the Mmp1 channel. Whole animal micrographs were captured with a Leica ZF10 stereomicroscope or Zeiss Z.1 Lightsheet microscope (see Supplementary Methods for details).

## Supplementary Material

Supplementary Information 2

Supplementary Data Set

## References

[bib1] Skau CT, Waterman CM. Specification of architecture and function of actin structures by actin nucleation factors. Annu Rev Biophys 2015; 44: 285–310. 26098516 10.1146/annurev-biophys-060414-034308 PMC6301004

[bib2] Stevenson RP, Veltman D, Machesky LM. Actin-bundling proteins in cancer progression at a glance. J Cell Sci 2012; 125: 1073–1079. 22492983 10.1242/jcs.093799

[bib3] Bravo-Cordero JJ, Hodgson L, Condeelis J. Directed cell invasion and migration during metastasis. Curr Opin Cell Biol 2012; 24: 277–283. 22209238 10.1016/j.ceb.2011.12.004 PMC3320684

[bib4] Krause M, Gautreau A. Steering cell migration: lamellipodium dynamics and the regulation of directional persistence. Nat Rev Mol Cell Biol 2014; 15: 577–590. 25145849 10.1038/nrm3861

[bib5] Olson EN, Nordheim A. Linking actin dynamics and gene transcription to drive cellular motile functions. Nat Rev Mol Cell Biol 2010; 11: 353–365. 20414257 10.1038/nrm2890 PMC3073350

[bib6] Grosse R, Copeland JW, Newsome TP, Way M, Treisman R. A role for VASP in RhoA-Diaphanous signalling to actin dynamics and SRF activity. EMBO J 2003; 22: 3050–3061. 12805219 10.1093/emboj/cdg287 PMC162139

[bib7] Sotiropoulos A, Gineitis D, Copeland J, Treisman R. Signal-regulated activation of serum response factor is mediated by changes in actin dynamics. Cell 1999; 98: 159–169. 10428028 10.1016/s0092-8674(00)81011-9

[bib8] Vartiainen MK, Guettler S, Larijani B, Treisman R. Nuclear actin regulates dynamic subcellular localization and activity of the SRF cofactor MAL. Science 2007; 316: 1749–1752. 17588931 10.1126/science.1141084

[bib9] Krause M, Leslie JD, Stewart M, Lafuente EM, Valderrama F, Jagannathan R et al. Lamellipodin, an Ena/VASP ligand, is implicated in the regulation of lamellipodial dynamics. Dev Cell 2004; 7: 571–583. 15469845 10.1016/j.devcel.2004.07.024

[bib10] Lafuente EM, van Puijenbroek AA, Krause M, Carman CV, Freeman GJ, Berezovskaya A et al. RIAM, an Ena/VASP and Profilin ligand, interacts with Rap1-GTP and mediates Rap1-induced adhesion. Dev Cell 2004; 7: 585–595. 15469846 10.1016/j.devcel.2004.07.021

[bib11] Law AL, Vehlow A, Kotini M, Dodgson L, Soong D, Theveneau E et al. Lamellipodin and the Scar/WAVE complex cooperate to promote cell migration *in vivo*. J Cell Biol 2013; 203: 673–689. 24247431 10.1083/jcb.201304051 PMC3840943

[bib12] Lyulcheva E, Taylor E, Michael M, Vehlow A, Tan S, Fletcher A et al. Drosophila pico and its mammalian ortholog lamellipodin activate serum response factor and promote cell proliferation. Dev Cell 2008; 15: 680–690. 19000833 10.1016/j.devcel.2008.09.020 PMC2691947

[bib13] Ginestier C, Cervera N, Finetti P, Esteyries S, Esterni B, Adelaide J et al. Prognosis and gene expression profiling of 20q13-amplified breast cancers. Clin Cancer Res 2006; 12: 4533–4544. 16899599 10.1158/1078-0432.CCR-05-2339

[bib14] Ross DT, Scherf U, Eisen MB, Perou CM, Rees C, Spellman P et al. Systematic variation in gene expression patterns in human cancer cell lines. Nat Genet 2000; 24: 227–235. 10700174 10.1038/73432

[bib15] Carmona G, Perera U, Gillett C, Naba A, Law AL, Sharma VP et al. Lamellipodin promotes invasive 3D cancer cell migration via regulated interactions with Ena/VASP and SCAR/WAVE. Oncogene 2016; 35: 5155–5169. 26996666 10.1038/onc.2016.47 PMC5031503

[bib16] Bild AH, Yao G, Chang JT, Wang Q, Potti A, Chasse D et al. Oncogenic pathway signatures in human cancers as a guide to targeted therapies. Nature 2006; 439: 353–357. 16273092 10.1038/nature04296

[bib17] Bennett D, Lyulcheva E, Cobbe N. Drosophila as a potential model for ocular tumors. Ocul Oncol Pathol 2015; 1: 190–199. 27172095 10.1159/000370155 PMC4847669

[bib18] Brumby AM, Richardson HE. Using *Drosophila melanogaster* to map human cancer pathways. Nat Rev Cancer 2005; 5: 626–639. 16034367 10.1038/nrc1671

[bib19] Choi C, Helfman DM. The Ras-ERK pathway modulates cytoskeleton organization, cell motility and lung metastasis signature genes in MDA-MB-231 LM2. Oncogene 2014; 33: 3668–3676. 23995792 10.1038/onc.2013.341

[bib20] Brumby AM, Richardson HE. scribble mutants cooperate with oncogenic Ras or Notch to cause neoplastic overgrowth in Drosophila. EMBO J 2003; 22: 5769–5779. 14592975 10.1093/emboj/cdg548 PMC275405

[bib21] Pagliarini RA, Xu T. A genetic screen in Drosophila for metastatic behavior. Science 2003; 302: 1227–1231. 14551319 10.1126/science.1088474

[bib22] Coussens LM, Werb Z. Inflammation and cancer. Nature 2002; 420: 860–867. 10.1038/nature01322 PMC2803035 12490959

[bib23] Srivastava A, Pastor-Pareja JC, Igaki T, Pagliarini R, Xu T. Basement membrane remodeling is essential for Drosophila disc eversion and tumor invasion. Proc Natl Acad Sci USA 2007; 104: 2721–2726. 17301221 10.1073/pnas.0611666104 PMC1815248

[bib24] Uhlirova M, Bohmann D. JNK- and Fos-regulated Mmp1 expression cooperates with Ras to induce invasive tumors in Drosophila. EMBO J 2006; 25: 5294–5304. 17082773 10.1038/sj.emboj.7601401 PMC1636619

[bib25] Martín-Blanco E, Gampel A, Ring J, Virdee K, Kirov N, Tolkovsky AM et al. *puckered* encodes a phosphatase that mediates a feedback loop regulating JNK activity during dorsal closure in Drosophila. Genes Dev 1998; 12: 557–570. 9472024 10.1101/gad.12.4.557 PMC316530

[bib26] Cordero JB, Macagno JP, Stefanatos RK, Strathdee KE, Cagan RL, Vidal M. Oncogenic Ras diverts a host TNF tumor suppressor activity into tumor promoter. Dev Cell 2010; 18: 999–1011. 20627081 10.1016/j.devcel.2010.05.014 PMC3175220

[bib27] Chotard C, Leung W, Salecker I. glial cells missing and gcm2 cell autonomously regulate both glial and neuronal development in the visual system of Drosophila. Neuron 2005; 48: 237–251. 16242405 10.1016/j.neuron.2005.09.019

[bib28] Read RD, Cavenee WK, Furnari FB, Thomas JB. A drosophila model for EGFR-Ras and PI3K-dependent human glioma. PLoS Genet 2009; 5: e1000374. 19214224 10.1371/journal.pgen.1000374 PMC2636203

[bib29] Bazigou E, Apitz H, Johansson J, Loren CE, Hirst EM, Chen PL et al. Anterograde Jelly belly and Alk receptor tyrosine kinase signaling mediates retinal axon targeting in Drosophila. Cell 2007; 128: 961–975. 17350579 10.1016/j.cell.2007.02.024

[bib30] Donlea JM, Ramanan N, Shaw PJ. Use-dependent plasticity in clock neurons regulates sleep need in Drosophila. Science 2009; 324: 105–108. 19342592 10.1126/science.1166657 PMC2850598

[bib31] Thran J, Poeck B, Strauss R. Serum response factor-mediated gene regulation in a Drosophila visual working memory. Curr Biol 2013; 23: 1756–1763. 24012317 10.1016/j.cub.2013.07.034

[bib32] Shekhar S, Pernier J, Carlier MF. Regulators of actin filament barbed ends at a glance. J Cell Sci 2016; 129: 1085–1091. 26940918 10.1242/jcs.179994

[bib33] Hansen SD, Mullins RD. Lamellipodin promotes actin assembly by clustering Ena/VASP proteins and tethering them to actin filaments. Elife 2015; 4: e06585. 10.7554/eLife.06585 PMC4543927 26295568

[bib34] Han Z, Li X, Wu J, Olson EN. A myocardin-related transcription factor regulates activity of serum response factor in Drosophila. Proc Natl Acad Sci USA 2004; 101: 12567–12572. 15314239 10.1073/pnas.0405085101 PMC515097

[bib35] Ohayon D, Pattyn A, Venteo S, Valmier J, Carroll P, Garces A. Zfh1 promotes survival of a peripheral glia subtype by antagonizing a Jun N-terminal kinase-dependent apoptotic pathway. EMBO J 2009; 28: 3228–3243. 19745814 10.1038/emboj.2009.247 PMC2771085

[bib36] Wu Y, Zhuang Y, Han M, Xu T, Deng K. Ras promotes cell survival by antagonizing both JNK and Hid signals in the Drosophila eye. BMC Dev Biol 2009; 9: 53. 19840402 10.1186/1471-213X-9-53 PMC2773777

[bib37] Andersen DS, Colombani J, Palmerini V, Chakrabandhu K, Boone E, Rothlisberger M et al. The Drosophila TNF receptor Grindelwald couples loss of cell polarity and neoplastic growth. Nature 2015; 522: 482–486. 25874673 10.1038/nature14298

[bib38] Friese MA, Steinle A, Weller M. The innate immune response in the central nervous system and its role in glioma immune surveillance. Onkologie 2004; 27: 487–491. 15585981 10.1159/000080371

[bib39] Logan MA, Freeman MR. The scoop on the fly brain: glial engulfment functions in Drosophila. Neuron Glia Biol 2007; 3: 63–74. 18172512 10.1017/S1740925X07000646 PMC2171361

[bib40] Shklover J, Mishnaevski K, Levy-Adam F, Kurant E. JNK pathway activation is able to synchronize neuronal death and glial phagocytosis in Drosophila. Cell Death Dis 2015; 6: e1649. 25695602 10.1038/cddis.2015.27 PMC4669801

[bib41] Macdonald JM, Doherty J, Hackett R, Freeman MR. The c-Jun kinase signaling cascade promotes glial engulfment activity through activation of draper and phagocytic function. Cell Death Differ 2013; 20: 1140–1148. 23618811 10.1038/cdd.2013.30 PMC3741495

[bib42] Chuang HN, van Rossum D, Sieger D, Siam L, Klemm F, Bleckmann A et al. Carcinoma cells misuse the host tissue damage response to invade the brain. Glia 2013; 61: 1331–1346. 23832647 10.1002/glia.22518 PMC3842117

[bib43] Rudrapatna VA, Bangi E, Cagan RL. A Jnk-Rho-Actin remodeling positive feedback network directs Src-driven invasion. Oncogene 2014; 33: 2801–2806. 23831567 10.1038/onc.2013.232

[bib44] Kulshammer E, Uhlirova M. The actin cross-linker Filamin/Cheerio mediates tumor malignancy downstream of JNK signaling. J Cell Sci 2013; 126: 927–938. 23239028 10.1242/jcs.114462

[bib45] Fernandez BG, Jezowska B, Janody F. Drosophila actin-capping protein limits JNK activation by the Src proto-oncogene. Oncogene 2014; 33: 2027–2039. 23644660 10.1038/onc.2013.155

[bib46] Balcer HI, Goodman AL, Rodal AA, Smith E, Kugler J, Heuser JE et al. Coordinated regulation of actin filament turnover by a high-molecular-weight Srv2/CAP complex, cofilin, profilin, and Aip1. Curr Biol 2003; 13: 2159–2169. 14680631 10.1016/j.cub.2003.11.051

[bib47] Didry D, Carlier MF, Pantaloni D. Synergy between actin depolymerizing factor/cofilin and profilin in increasing actin filament turnover. J Biol Chem 1998; 273: 25602–25611. 9748225 10.1074/jbc.273.40.25602

[bib48] Nawaz S, Sanchez P, Schmitt S, Snaidero N, Mitkovski M, Velte C et al. Actin filament turnover drives leading edge growth during myelin sheath formation in the central nervous system. Dev Cell 2015; 34: 139–151. 26166299 10.1016/j.devcel.2015.05.013 PMC4736019

[bib49] Pinheiro EM, Xie Z, Norovich AL, Vidaki M, Tsai LH, Gertler FB. Lpd depletion reveals that SRF specifies radial versus tangential migration of pyramidal neurons. Nat Cell Biol 2011; 13: 989–995. 21785421 10.1038/ncb2292 PMC3149714

[bib50] Esnault C, Stewart A, Gualdrini F, East P, Horswell S, Matthews N et al. Rho-actin signaling to the MRTF coactivators dominates the immediate transcriptional response to serum in fibroblasts. Genes Dev 2014; 28: 943–958. 24732378 10.1101/gad.239327.114 PMC4018493

[bib51] Salvany L, Muller J, Guccione E, Rorth P. The core and conserved role of MAL is homeostatic regulation of actin levels. Genes Dev 2014; 28: 1048–1053. 24831700 10.1101/gad.237743.114 PMC4035534

[bib52] Ziv-Av A, Taller D, Attia M, Xiang C, Lee HK, Cazacu S et al. RTVP-1 expression is regulated by SRF downstream of protein kinase C and contributes to the effect of SRF on glioma cell migration. Cell Signal 2011; 23: 1936–1943. 21777672 10.1016/j.cellsig.2011.07.001

[bib53] Petryszak R, Keays M, Tang YA, Fonseca NA, Barrera E, Burdett T et al. Expression Atlas update-an integrated database of gene and protein expression in humans, animals and plants. Nucleic Acids Res 2016; 44: D746–D752. 26481351 10.1093/nar/gkv1045 PMC4702781

[bib54] Ciurciu A, Duncalf L, Jonchere V, Lansdale N, Vasieva O, Glenday P et al. PNUTS/PP1 regulates RNAPII-mediated gene expression and is necessary for developmental growth. PLoS Genet 2013; 9: e1003885. 24204300 10.1371/journal.pgen.1003885 PMC3814315

[bib55] Kurucz E, Markus R, Zsamboki J, Folkl-Medzihradszky K, Darula Z, Vilmos P et al. Nimrod, a putative phagocytosis receptor with EGF repeats in Drosophila plasmatocytes. Curr Biol 2007; 17: 649–654. 17363253 10.1016/j.cub.2007.02.041

[bib56] Allan C, Burel JM, Moore J, Blackburn C, Linkert M, Loynton S et al. OMERO: flexible, model-driven data management for experimental biology. Nat Methods 2012; 9: 245–253. 22373911 10.1038/nmeth.1896 PMC3437820

