# Supplementary figures and images for: MRL proteins cooperate with activated Ras in glia to drive distinct oncogenic outcomes
Source: Oncogene. 2017 Mar 27;36(30):4311–22. doi: 10.1038/onc.2017.68 (PMC5537612; doi:10.1038/onc.2017.68)

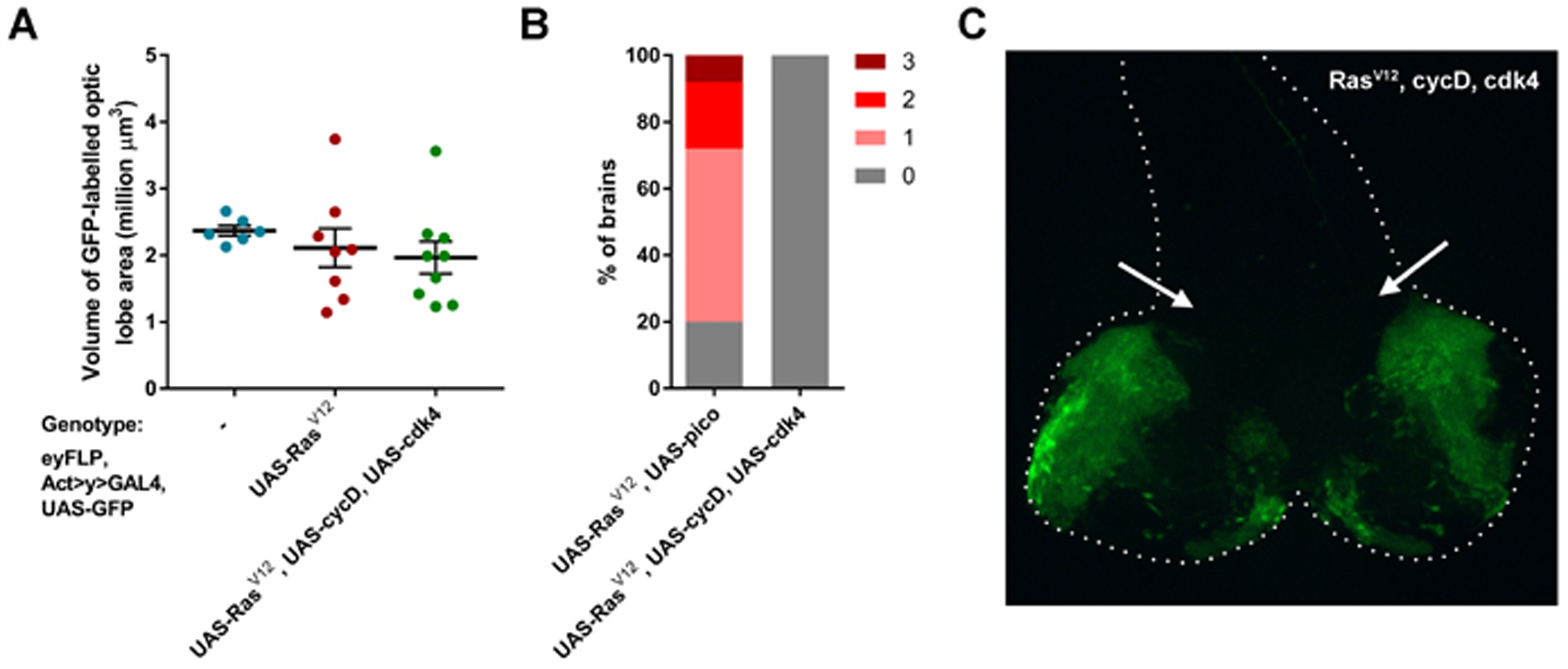

Supplement: Supplementary Figure 1 [file onc201768x1.tif]

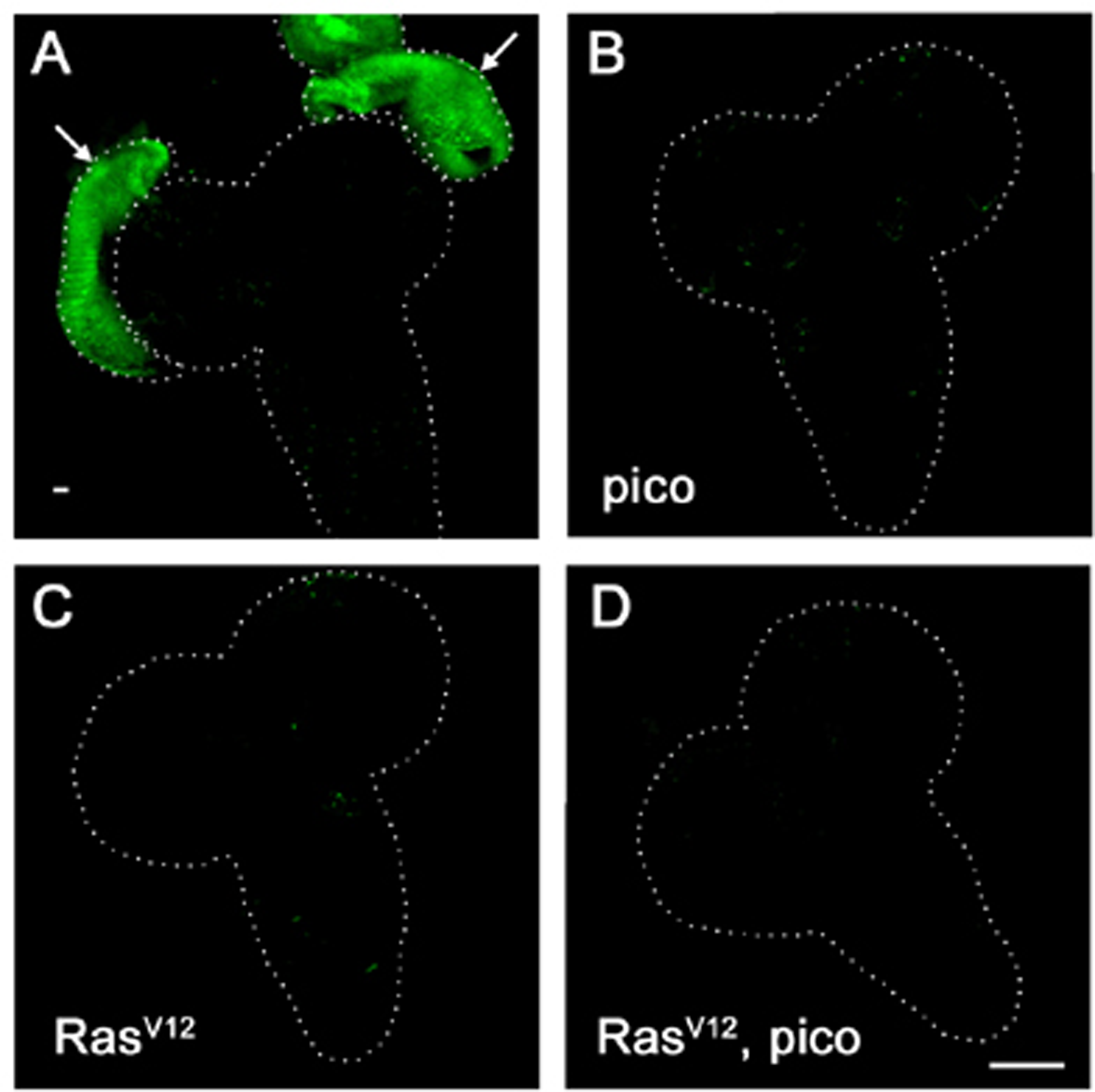

Supplement: Supplementary Figure 2 [file onc201768x2.tif]

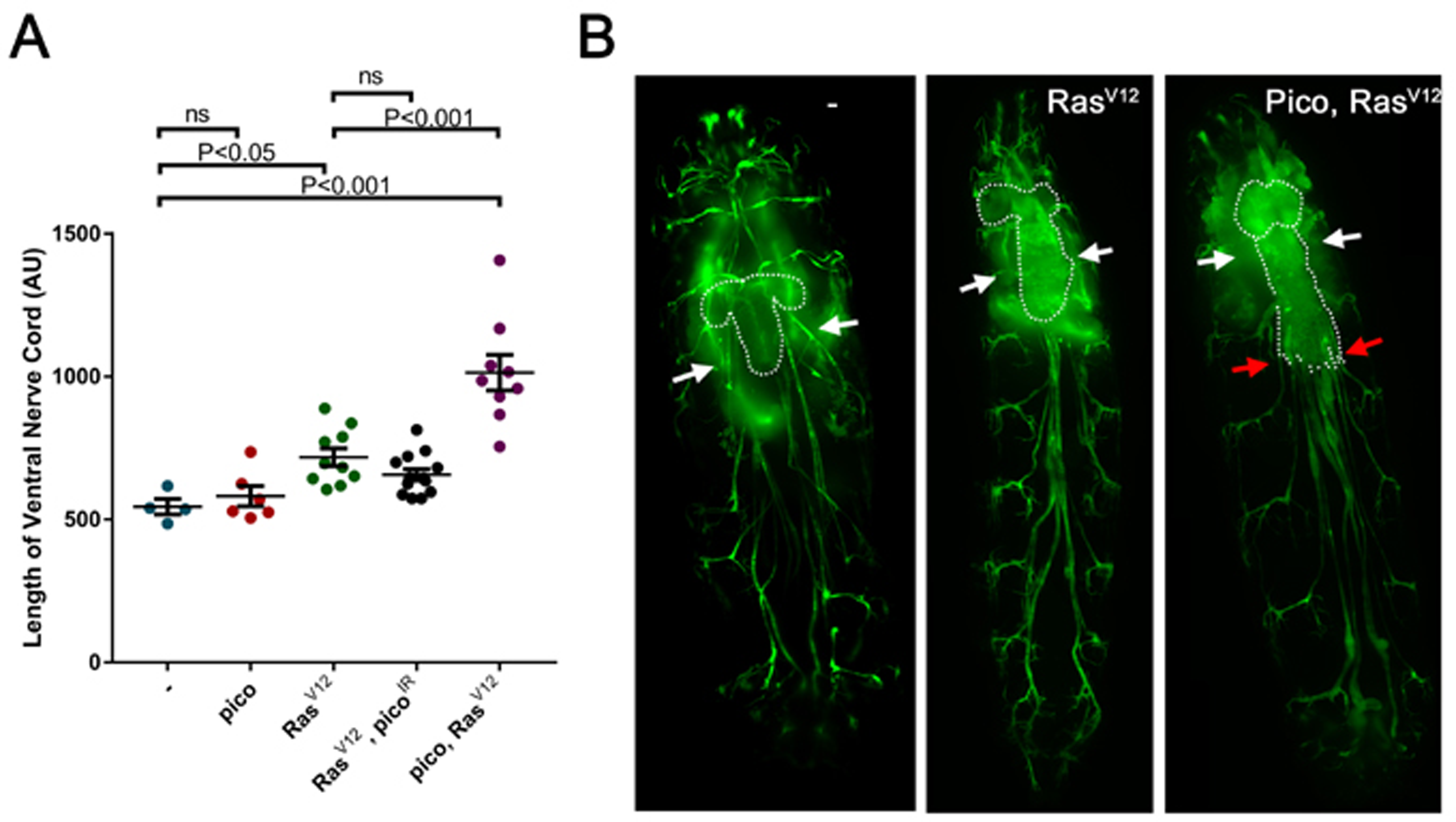

Supplement: Supplementary Figure 3 [file onc201768x3.tif]
