# Supplementary material for: MRL proteins cooperate with activated Ras in glia to drive distinct oncogenic outcomes
Source: Oncogene. 2017 Mar 27;36(30):4311–22. doi: 10.1038/onc.2017.68 (PMC5537612; doi:10.1038/onc.2017.68)
Supplement: Supplementary Information 1 [file onc201768x4.pdf]

Genotypes

w, eyFLP; A>y>GAL4, UAS-GFP/+; UAS-HM-pico/+ (Fig.1,2,5)
w, eyFLP; A>y>GAL4, UAS-GFP/+; UAS-Ras^{V12}*/+*
w, eyFLP; A>y>GAL4, UAS-GFP/+; UAS-Ras^{V12}*, UAS-HM-pico/+*
w, eyFLP; A>y>GAL4, UAS-GFP/+; UAS-Ras^{V12}*, UAS-hLpd/+*

w, eyFLP; A>y>GAL4, UAS-GFP/+; UAS-HM-pico/puc-lacZ (Fig.3)
w, eyFLP; A>y>GAL4, UAS-GFP/+; UAS-Ras^{V12}*/puc-lacZ*
w, eyFLP; A>y>GAL4, UAS-GFP/+; UAS-Ras^{V12}*, UAS-HM-pico/puc-lacZ*
w, eyFLP/UAS-DN-Bsk; A>y>GAL4, UAS-GFP/+; UAS-HM-pico/puc-lacZ
w, eyFLP/UAS-DN-Bsk; A>y>GAL4, UAS-GFP/+; UAS-Ras^{V12}*/puc-lacZ*
w, eyFLP/UAS-DN-Bsk; A>y>GAL4, UAS-GFP/+; UAS-Ras^{V12}*, UAS-HM-pico/puc-lacZ*

w, eyFLP; +/+; UAS-Ras^{V12}*, UAS-HM-pico/A>CD2>GAL4,UAS-GFP* (Fig.4)
w, eyFLP; eiger^{3}*/eiger*^{3}*; UAS-Ras*^{V12}*, UAS-HM-pico/A>CD2>GAL4,UAS-GFP*

w, eyFLP; +/+; UAS-Ras^{V12}*, UAS-HM-pico/A>CD2>GAL4, UAS-GFP* (Fig.6)
w, eyFLP; +/repo-GAL80; UAS-Ras^{V12}*, UAS-HM-pico/A>CD2>GAL4, UAS-GFP*

w, eyFLP; A>y>GAL4, UAS-GFP/+ (Fig.7)
w, eyFLP; A>y>GAL4, UAS-GFP/+; UAS-Ras^{V12}*, UAS-HM-pico/+*
w, eyFLP; A>y>GAL4, UAS-GFP/+; UAS-Ras^{V12}*, UAS-HM-pico*$^{RA\text{-}PH}$
w, eyFLP; UAS-ena/+; A>CD2>GAL4,UAS-GFP/+
w, eyFLP; UAS-ena/+; UAS-Ras^{V12}*/A>CD2>GAL4,UAS-GFP*
w, eyFLP; UAS-ena/+; UAS-Ras^{V12}*, UAS-HM-pico/A>CD2>GAL4,UAS-GFP*
w, eyFLP; UAS-chic/+; A>CD2>GAL4,UAS-GFP/+
w, eyFLP; UAS-chic/+; UAS-Ras^{V12}*/A>CD2>GAL4,UAS-GFP*
w, eyFLP; UAS-chic/+; UAS-Ras^{V12}*, UAS-HM-pico/A>CD2>GAL4,UAS-GFP*
w, eyFLP; UAS-scar/+; A>CD2>GAL4,UAS-GFP/+
w, eyFLP; UAS-scar/+; UAS-Ras^{V12}*/A>CD2>GAL4,UAS-GFP*
w, eyFLP; UAS-mal-d/+; A>CD2>GAL4,UAS-GFP/+
w, eyFLP; UAS-mal-d/+; UAS-Ras^{V12}*/A>CD2>GAL4,UAS-GFP*
w, eyFLP; UAS-mal-d/+; UAS-Ras^{V12}*, UAS-HM-pico/A>CD2>GAL4,UAS-GFP*
w, eyFLP; A>y>GAL4, UAS-GFP/+; UAS-Ras^{V12}*, UAS-HM-pico/UAS-mal*^{DN}
w, eyFLP; ena^{210}*/+; UAS-Ras*^{V12}*, UAS-HM-pico/A>CD2>GAL4,UAS-GFP*
w, eyFLP; chic^{05205}*/+; UAS-Ras*^{V12}*, UAS-HM-pico/A>CD2>GAL4,UAS-GFP*
w, eyFLP; UAS-Scar^{IR}*/+; UAS-Ras*^{V12}*, UAS-HM-pico/A>CD2>GAL4,UAS-GFP*

We consider *A>y>GAL4* and *A>CD2>GAL4* elements used in this study to be comparable because: i) neither were able to promote invasion of *Ras*^{V12}-expressing cells into the ventral nerve cord; ii) both elements were capable of driving invasion of *Ras*^{V12} *Pico*-expressing cells, to an equivalent extent, see Fig.7.

w, eyFLP; A>y>GAL4, UAS-GFP/+; UAS-Ras^{V12}*/+* (Fig.S1)

w, eyFLP; A>y>GAL4, UAS-GFP/+; UAS-Ras^{V12}*/UAS-CycD, UAS-Cdk4*

w, ey(3.5)FLP; A>y>GAL4, UAS-GFP/+; (Fig.S2)

w, ey(3.5)FLP; A>y>GAL4, UAS-GFP/+; UAS-HM-pico/+

w, ey(3.5)FLP; A>y>GAL4, UAS-GFP/+; UAS-Ras^{V12}*/+*

w, ey(3.5)FLP; A>y>GAL4, UAS-GFP/+; UAS-Ras^{V12}*, UAS-HM-pico/+*

UAS-mCD8-GFP/+; repo-GAL4/+ (Fig.S3)

UAS-mCD8-GFP/+, repo-GAL4/ UAS-Ras^{V12}

UAS-mCD8-GFP/+; repo-GAL4/ UAS-HM-pico

UAS-mCD8-GFP/+; repo-GAL4/ UAS-Ras^{V12}*, UAS-HM-pico*

UAS-mCD8-GFP/+; repo-GAL4/ UAS-Ras^{V12}*, UAS-picoIR (line 4)*
