# Supplementary material for: MRL proteins cooperate with activated Ras in glia to drive distinct oncogenic outcomes
Source: Oncogene. 2017 Mar 27;36(30):4311–22. doi: 10.1038/onc.2017.68 (PMC5537612; doi:10.1038/onc.2017.68)
Supplement: Supplementary Information 2 [file onc201768x5.pdf]

Supplementary methods

Lightsheet microscope image acquisition and processing. mCD8::GFP third Instar larvae were collected at the bottom of a 1.5 ml tube and anesthetized by chloroform vapors for 5 minutes. For embedding, 1.5% (w/v in PBS) low-melting-point liquid agarose was added to the tube and larvae were drawn into a glass capillary with plungers and extruded after polymerization into the acquisition chamber. A suitable number of acquisition fields were captured to cover the size of an entire larva. For each field, about a hundred of Z-projected images were captured on Zeiss Lightsheet Z.1 Microscope equipped with 488 nm laser using a 20x objective, and maximum intensity projection images were generated using Zen 2011 software (Zeiss). Reconstructed images of whole larvae were obtained using a Stitching plugin in Fiji software[1].

1. S. Preibisch, S. Saalfeld, P. Tomancak. Globally optimal stitching of tiled 3D microscopic image acquisitions. *Bioinformatics* 2009; **25**:1463-1465.
